# Grapevine Wood-Degrading Activity of *Fomitiporia mediterranea* M. Fisch.: A Focus on the Enzymatic Pathway Regulation

**DOI:** 10.3389/fmicb.2022.844264

**Published:** 2022-03-18

**Authors:** Andrea Pacetti, Samuele Moretti, Célia Perrin, Eric Gelhaye, Evi Bieler, Hanns-Heinz Kassemeyer, Laura Mugnai, Sibylle Farine, Christophe Bertsch

**Affiliations:** ^1^Laboratoire Vigne Biotechnologies et Environnement UR-3991, Université de Haute Alsace, Colmar, France; ^2^Department of Agricultural, Food, Environmental and Forestry Science and Technology (DAGRI), Plant Pathology and Entomology Section, University of Florence, Florence, Italy; ^3^Faculté des Sciences et Technologies Boulevard des Aiguillettes, UMR IAM - Université de Lorraine, Vandoeuvre-lès-Nancy, France; ^4^Swiss Nanoscience Institute (SNI) – Nano Imaging, University of Basel, Basel, Switzerland; ^5^State Institute for Viticulture, Plant Pathology & Diagnostics, Freiburg, Germany; ^6^Faculty of Biology, Plant Biomechanics Group and Botanic Garden, Albert-Ludwigs-Universität Freiburg, Freiburg im Breisgau, Germany

**Keywords:** decay, SEM, cellulose, lignin, grapevine trunk diseases, Esca

## Abstract

*Fomitiporia mediterranea* is a *Basidiomycetes* fungus associated with some of the Esca complex diseases and responsible for decay in grapevine wood. Its role in the onset of foliar symptoms has recently been reconsidered, mainly after evidence showing a reduction in foliar symptom expression after removal of rotten wood. The study of its degradation pathways has already been approached by other authors, and with this study much information is consolidated. A microscopic observation of degraded wood provides a first approach to the characterization of *F. mediterranea* modalities of wood cellular structure degradation. The decay of grapevine wood was reproduced *in vitro*, and the measurement of each wood-forming polymer loss highlighted characteristics of *F. mediterranea* common to selective white rot and showed how fungal strain and vine variety are factors determining the wood degradation. All these observations were supported by the analysis of the laccase and manganese peroxidase enzyme activity, as well as by the expression of the genes coding 6 putative laccase isoforms and 3 manganese peroxidase isoforms, thereby highlighting substantial intraspecific variability.

## Introduction

Grapevine trunk diseases (GTDs) are considered “the biotic stress of the century” for the grapevine (Songy et al., 2019), with an increasing economic impact in all grape-growing countries. Among them, the Esca complex of diseases (ECDs) is a huge problem in Europe, where disease incidence (measured by foliar symptoms and vine death) reached “increasing” and/or “worrying” levels in several regions of Italy, France, and Spain (Guérin-Dubrana et al., 2019). This has led to huge economic losses (up to 1 billion euros per year estimated by the French Wine Institute, IFV), justifying the interest in increasing research and knowledge gathering in that area (Claverie et al., 2020). The Esca complex is currently considered a complex of different diseases, characterized by several different symptoms. Even though a quite complex microbiota has been recently shown to be involved in its onset (Bruez et al., 2020), the diseases of the complex are usually associated with the Ascomycota species including *Phaeomoniella chlamydospora* (Pch) (Crous and Gams, 2000), *Phaeoacremonium minimum* (Pmin) (Gramaje et al., 2015), and, in Europe, the basidiomycete *Fomitiporia mediterranea* (Fmed), while in other grapevine-growing regions other *Fomitiporia* species can be identified (Fischer, 2002, 2006; Del Frari et al., 2021; Moretti et al., 2021). Pch and Pmin are considered to be responsible for the “phaeotracheomycotic complex” (brown wood streaking; Bertsch et al., 2013), while Fmed is a white-rot agent responsible for wood degradation and decay, historically associated with “Esca” (Gard, 1922) and “Esca proper” (Surico, 2009). Despite Esca being described as a complex of diseases, epidemiological analysis mostly relies on the description of the typical striped leaf symptoms (characterizing grapevine leaf stripe disease following Surico, 2009) and vine apoplexy (sudden wilting of part or the whole vine), given the difficulty of assessing internal wood symptoms in standing vines, i.e., brown or black wood streaking, central or sectorial necrosis, and white rot. However, recently new interest has arisen in the activity and role of the white-rot agent *Fomitiporia mediterranea* (Moretti et al., 2021), on the basis of increasing reports on the direct effect on symptom development following white-rot elimination (Thibault, 2015; Dal, 2020; Cholet et al., 2021; Pacetti et al., 2021). Studies on microbiota composition in wood rot and grapevine leaf stripe disease (GLSD) symptomatic vines (Del Frari et al., 2019; Bruez et al., 2020; Pacetti et al., 2021) are shedding new light on the historically debated—and never fully clarified—relationship between white rot and GLSD-foliar symptom expression (Calzarano and Di Marco, 2007; Maher et al., 2012). Wood is the major structure that gives trees and other woody plants and vines stability for upright growth and maintains the water supply from the roots to all other plant tissues. Woody plant cell walls consist mainly of a lignocellulose complex, which is composed of cellulose, hemicellulose, and lignin heteropolymers, organized in different ratios depending on the woody plant species (Lundell et al., 2010). Lignin generally represents the second most abundant biopolymer of the plant cell wall in hardwood and softwood species (Pettersen, 1984; Howard et al., 2003; Chen et al., 2014), while in grapevine wood it represents the less abundant one in accordance with its liana morphological characteristics (Agrelli et al., 2009). Lignin also has an important defensive role in plant–pathogen interaction (Weng and Chapple, 2010), especially in host resistance against white-rot agents (Schwarze, 2007). Despite its abundance in woody plants, lignin is neither an energy nor a carbon source for fungi if available alone, but, as lignin in the plant cell wall is constitutively merged with carbohydrates, the *in vivo* degradation by a concert of enzyme activity could lead to complete digestion (Kirk et al., 1976; Chen et al., 2014). The processes of depolymerization of cellulose and lignin are interrelated, and they can even boost each other (Westermark and Eriksson, 1974a,b, 1975). The fact remains that to access cell wall carbohydrates, a lignin bypass or degradation mechanism is required (Cragg et al., 2015). Historically, white-rot degradation was described as the result of complete lignin, cellulose, and hemicellulose mineralization driven by extracellular enzymes (Blanchette, 1991). Many families of degrading enzymes are involved in the degradation of white-rot wood such as carbohydrate active enzymes (CAZymes) for cellulose and hemicelluloses or laccases and Class II peroxidases (PODs) for lignin (Daniel, 2014), as well as several auxiliary active (AA) redox enzymes, a class of enzymes currently associated with CAZymes and Class II PODs that sustain ligninolytic enzyme activity, with results present in white-rot fungi (Levasseur et al., 2013). Nevertheless, a comparative genomic study based on CAZymes of 31 fungal species showed that the prevailing paradigm of white versus brown rot does not capture the diversity of wood-decay mechanisms in *Basidiomycetes* (Riley et al., 2014). Fmed is nowadays considered a white-rot agent (Fischer, 2002), and its degrading secretome has been investigated by many authors (Mugnai et al., 1999; Bruno and Sparapano, 2006; Abou-Mansour et al., 2009; Schilling et al., 2021). Besides several identified or targeted enzymes belonging to the CAZyme pool which degrade cellulose (Mugnai et al., 1999; Bruno and Sparapano, 2006; Riley et al., 2014), as a white-rot species Fmed has all the main enzymes described to fully mineralize lignin, enzymes that are involved in the pathogenicity process, specifically (i) Class II PODs, such as manganese peroxidases (MnP; EC 1.11.1.13), and (ii) laccases (EC 1.10.3.2; p-diphenol:di-oxygen oxidoreductases). Three MnP genes were characterized in the Fmed genome, *Fmmnp1*, *Fmmnp2*, and *Fmmnp3* (Morgenstern et al., 2010). Although some authors have highlighted the lignin peroxidase (LiP) activity of Fmed *in vitro* (Cloete et al., 2015b), a comprehensive study based on genome sequencing of wood-degrading fungi reported the absence of LiP (EC1.11.1.14) genes in the Fmed genome (Floudas et al., 2012). Abou-Mansour et al. (2009) purified a typical fungal 60-kDa laccase from some Fmed isolates, even though complete lignin degradation was not achieved by these proteins alone. Currently, the role of laccases in pathogenicity itself is still being debated (Giardina et al., 2010; Singh Arora and Kumar Sharma, 2010). As suggested in a recent review on Fmed (Moretti et al., 2021), studying the pathogenicity factors involved in grapevine wood rot, in order to fill the gaps in information concerning the biomolecular process of white rot, could eventually furnish keys to contribute to the interpretation of the etiology of the leaf stripe symptoms. For that purpose, we first performed a visual analysis of cell wall degradation by epi-fluorescence microscopy, on naturally Fmed-infected grapevine wood, highlighting the degradation of all components of the wood cell wall due to the enzyme activity. In order to characterize this activity, we focused on the lignin-degrading enzymes, the ones that could be responsible not only for causing the white rot itself but also possibly involved in the “by-product of wood degradation” theory on the appearance of striped leaves symptoms postulated by Mugnai et al. (1999) and investigated by other authors (Agrelli et al., 2009; Amalfitano et al., 2011). In particular, we investigated *in vitro* (i) the enzyme activity and kinetics of ligninolytic enzymes; (ii) their molecular regulation; and (iii) the residual wood polymer after fungal degradation.

## Materials and Methods

### Epi-FM on Naturally Infected Wood

For microscopic analysis, trunks of grapevines of cv. Riesling (Kober5bb rootstock), planted in 1997, and cv. Sauvignon blanc (SO4 rootstock), planted in 2009, were used. The sampling sites were neighboring vineyards on gently west-sloping slopes on loess and loess loam (subsoil Tertiary marls, limestones, and sandstones) in the Batzenberg area of southwestern Germany, south of Freiburg (47.97°N, 7.75 E and 47.95°N, 7.74 E). Using a band saw, longitudinal and transverse sections were taken from the trunks, from which segments of 10 mm × 5 mm × 5 mm from lesions with white rot were excised. The samples were fixed in glutaric aldehyde for more than 24 h and, after being washed three times in deionized water, dehydrated in an increasing concentration of isopropyl alcohol. After embedding in methacrylate resin, semi-thin sections of 3 and 1 μm were prepared with a rotary microtome (LEICA 2065 and LEICA 2044). For further processing, the resin was removed from the sections by rinsing overnight in isopropyl alcohol. Next, the specimens that had been fixed on glass slides were stained in a programmable slide stainer (ZEISS HMS TM Series, Carl Zeiss AG, Oberkochen, Germany) with 2% safranin and 1% acriflavine (12 h), 1% acid yellow (30 min), and 1% methylene blue (5 min) and embedded in Eukitt (O. Kindler, Freiburg, Germany). For epi-fluorescence microscopy (epi-FM), the slides were stained with acridine orange. The microscopic analyses were carried out with a light (bright-field) and epi-fluorescence microscope (ZEISS Axio Imager Z1) equipped with the optical sectioning system Apotome 2 for structural illumination and a digital imaging system (ZEISS Axiocam MR35, ZEN 2.6 pro image processing software by Carl Zeiss AG, Oberkochen, Germany). FM-3D image visualization of the samples stained with acridine orange was performed with the filter combination 38 HE, excitation 460-488 nm, emission 500-557 nm.

### Fungal Isolation From Wood Samples and Identification

To attribute microscopic observations to certain fungal species, classic isolations were made by sampling fragments of fungal-colonized wood, contiguous to the tissue sampled for microscopic analysis. In order to establish which fungal species were responsible for the observed wood alterations, isolations were made from 50 wood fragments. The wood fragments were placed on PDA (Potato Dextrose Agar), with streptomycin sulfate at 1 g*l^–1^, for 30 days at 28°C in the dark, and each colony developed on the medium was transferred to a fresh PDA plate for identification. The morphology of the isolated fungi was studied by optical microscopy.

### *Fomitiporia mediterranea* Strains

Four strains of *Fomitiporia mediterranea* (Table 1) from a mycological collection, different from that isolated for microscopy, were tested for both *in vitro* and enzyme activity assay. The identity of selected strains was confirmed by culture morphology and ITS sequence data.

**TABLE 1:** *Fomitiporia mediterranea* strains used in laboratory experiments in this study.

Strain	Location	Host	Isolation date
LR71	Vendargues (34), FR	*V. vinifera* cv. Alphonse Lavallée	1996
LR124	Villeneuve les Maguelone (34), FR	*V. vinifera* cv. Carignan	1996
PHCO36	Saint-Preuil (16), FR	*V. vinifera* cv. Ugni blanc	1996
235.01	Riotorto (Livorno), IT	*Olea europaea L.*	2003

### *Fomitiporia mediterranea* Solid and Liquid Cultures

To study enzyme activity regulation and related gene expression, liquid cultures and solid cultures were set up, respectively. Solid cultures of the selected strains of Fmed were grown at 28°C in the dark on 90-mm petri dishes of Eriksson and Pettersson medium (Eriksson and Pettersson, 2005) (E&P) solidified by adding 15 mg of agar per liter. Liquid cultures were prepared using 125 ml of E&P medium supplemented with 0.625 g of cellulose and 1.25 g of *Vitis vinifera* (cv. Gewurztraminer) wood sawdust and inoculated with a section (1/4) of 20-day-old solid culture in a 250-ml flask. Liquid cultures were grown for 10 days at 28°C, shaken at 150 rpm in the dark. Three biological replicates per strain were tested. Three technical replicates of 10 ml of each sample were sampled and filtered by 4-stage filtration: 50 μm Nilex sheet and 1-, 0.45-, and 0.2-μm regenerated cellulose membrane filters. The whole solid mass of each technical replicate was separated from the liquid, dried at 110°C for 48 h, and weighed. The protein content of filtered liquid (hereinafter secretome) was defined by spectrophotometry using a NanoDrop (BioSpec-nano, Shimadzu, Kyoto, Japan) and assuming that 1 OD corresponded to a concentration of 1 mg/ml. The culture secretome was diluted to a final concentration of 0.2 mg/ml for enzymatic assays.

### Determination of Residual Wood Polymer After Fungal Degradation

**FIGURE 1:**
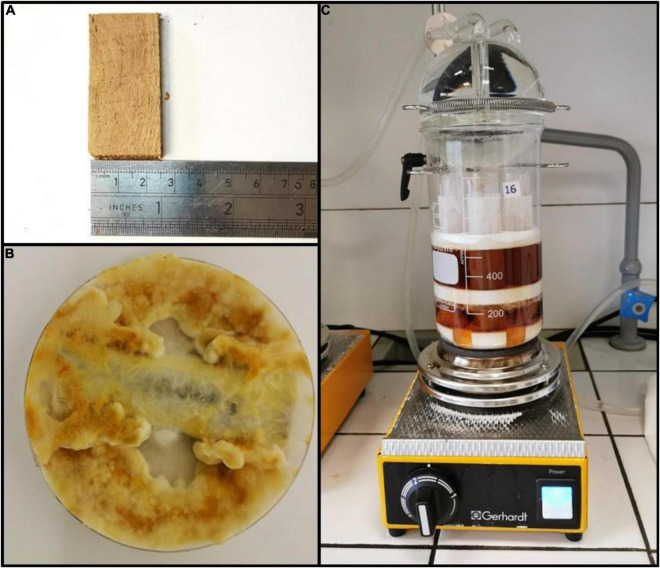
**(A)** Grapevine wood block before fungal degradation. **(B)** Wood block incubated with Fmed in a petri dish after complete wood colonization. **(C)** Plastic mesh-filter bag with spacers during the detergent bath for neutral detergent fiber (NDF), acid detergent fiber (ADF), and acid detergent lignin (ADL) determination.

To estimate the degradation of each grapevine wood polymers caused by Fmed, cv. Teroldego and cv. Gewurztraminer vine wood blocks were used for *in vitro* assays. Cultivar Teroldego was chosen as a variety rarely showing the leaf foliar symptoms of the Esca complex, while cv. Gewurztraminer was selected as a highly symptomatic variety (Grosman and Doublet, 2012; Bottura, 2018). For each variety, 3 blocks of wood were shaped to 0.5 cm × 5 cm × 2.5 cm (Figure 1A) and degraded for 30 and 90 days by 2 of the selected strains of Fmed (LR124 and PHCO36). Each block weighed 10 g with a variation of less than 0.5 g, and the wood did not show necrosis or discoloration. The wood blocks were dried and sterilized at 110°C for 48 h in the oven and then placed in petri dishes on 20 ml of solid E&P medium with four 6-mm plugs from each Fmed 20-day-old culture on E&P (Figure 1B). Three more blocks for each variety were also sterilized and used as controls without inoculation. To confirm that the wood degradation activity was carried out by the target fungus, samples were analyzed by SEM before and after colonization. In addition, the grapevine undegraded wood polymer composition was determined on wood blocks not inoculated with Fmed strains and, in order to compare the resulting profiles to better known soft- and hardwood profiles, also three samples of beech (*Fagus sylvatica* L.) and three samples of spruce (*Picea abies* L., H. Karst.), a hardwood and a softwood, respectively, were analyzed. After degradation, the fungal mycelium was scraped off from the surface of wood blocks and the residual wood was dried again as before the inoculation. After drying, the wood blocks were lyophilized, then frozen with liquid nitrogen and pulverized using a mixer mill (Retsch MM 400, Retsch GmbH, Haan, Germany). The wood powder obtained was digested using detergents and acid solutions in a 3-step protocol. One gram (± 0.01 g) of the powdered samples was placed in a plastic mesh-filter bag (Fibrebags ADF, C. Gerhardt GmbH & Co.) with a spacer and subjected to 3 consecutive baths (Figure 1C). After each bath, samples were dried in order to measure neutral detergent fiber (NDF), acid detergent fiber (ADF), and acid detergent lignin (ADL) (Van Soest and McQueen, 1973). The neutral detergent solution was composed of sodium dodecyl sulfate (30 g/l), sodium EDTA (18.61 g/l), sodium phosphate monobasic (4.56 g/l), sodium tetraborate decahydrate (6.81 g/l), and triethylene glycol (10 ml/l). The pH of the solution was adjusted to between 6.9 and 7.1. Samples were immersed in this boiling solution for 1 h and washed 5 times in hot water to eliminate the detergent solution. Then, the samples were dried at 105°C overnight and weighed to determine the NDF fraction. The second solution was composed of 20 g of cetyltrimethylammonium bromide (CTAB) dissolved in 11 ml of sulfuric acid (0.5 mol/l). Samples were immersed in this boiling solution for 1 h and washed 5 times in hot water to eliminate the acidic solution. Then, the samples were dried again at 105°C overnight and weighed to determine the ADF fraction. The third solution was composed of 72% sulfuric acid. The samples were then immersed in this solution for 3 h at room temperature and washed 5 times in hot water to eliminate the acidic solution. Finally, the samples were dried one last time at 105°C overnight and weighed to determine the ADL fraction.

### Scanning Electron Microscopy on *in vitro* Incubated Wood Blocks

Scanning electron microscopy (SEM) was performed on incubated grapevine wood blocks (for 30 and 90 days) to confirm that the degradation was carried out by the inoculated Fmed strains: the surface of the dried samples was ground and polished with the Leica EM TXP Target Surfacing System. After sputtering with 20 nm gold, the wood structure was analyzed using the Philips XL30 ESEM environmental scanning electron microscope with an SE detector at an accelerating voltage of 5-10 kV. The image processing was done with DISS5 Software from REM-X GmbH Bruchsal (Germany).

### Enzyme Activity of Fmed Secretome

For the evaluation of enzyme activity, the liquid culture experimental setup was used. Laccase activity was indirectly determined by oxidation of ABTS (2.2′-azino-bis(3-ethylbenzothiazoline-6-sulfonic acid)) to ABTS^+^. Oxidized ABTS turns color from transparent to green and can be measured by spectrophotometry. The absorbance was measured at 420 nm, every 20 s for 250 s from the addition of 25 μl of ABTS (2 mM) to a mix of 25 μl of sodium acetate buffer (100 mM, pH 4.5) and 50 μl of liquid culture secretome (0.2 mg/ml). Absorption values were converted into enzyme activity *via* the extinction coefficient of ABTS (ε_420_ = 3.6 10^4^ M^–1^ * cm^–1^) (Bourbonnais et al., 1995). According to Mathieu et al. (2013), MnP activity assay was based on the reaction of MBTH (3-methyl-2-benzothiazolinonehydrazone hydrochloride) and DMAB (para-dimethylaminobenzaldehyde) catalyzed by PODs in the presence of H_2_O_2_. The reaction allows the formation of indamine, a purple-colored compound which absorbs at 590 nm.

Tests were carried out in two steps, using two different reagent solutions. The common reagent mix contained 0.5 ml of DMAB (50 mM) and 0.5 ml MBTH (1 mM) in a buffer mix of 5 ml of sodium lactate (100 mM) and sodium succinate buffer (100 mM) adjusted to pH 4.5. For the first reaction, 1 ml of MnSO_4_ (1 mM)—which is required for manganese-dependent PODs—was added to the common reagent mix (solution A) to measure the specific MnP activity. Secondly, the total POD activity was assessed adding 1 ml EDTA (2 mM) to the common reagent mix instead of manganese sulfate (solution B). MnP activity was calculated by subtracting enzyme activity measured with solution B from that measured with solution A. Since some studies exclude the presence of LiP-encoding genes in the Fmed genome, the enzymatic activity of these enzymes has not been tested (Floudas et al., 2012). Absorbance measures were carried out on a reaction mixture prepared with 140 μl of reacting solution, 50 μl of liquid culture secretome (0.2 mg/ml), and 10 μl of H_2_O_2_ (1 mM), added to activate the reaction. Absorbance was then converted into enzyme activity *via* the extinction coefficient (32,000 M^–1^ * cm^–1^). The relative activities of each enzyme were determined by spectrophotometry, and all reactions were driven at 28°C maintaining the reaction mix in a 96-well microplate (flat-bottom F96 immuno plate, Nunc^®^, Roskilde, Denmark), using a filter-based plate reader (Tristar 2, Berthold Technologies GmbH & Co. KG, Bad Wildbad, Germany). Two types of control were run by using heat-inactivated samples and H_2_O_2_-free mix. All specific enzyme activity is expressed as specific activity by U/mg unit, where 1 U = 1 μmol of substrate oxidized per minute; the enzyme activity was measured during assays, and obtained U/l was then divided by the protein concentration of the secretome.

### Total RNA Isolation and cDNA Synthesis

Total RNA isolation was performed in 3 technical replicates: 0.2 g of fresh mycelium scraped from each sample of E&P-solid cultures, made by scraping fresh mycelium from the dish surface, frozen in liquid nitrogen, ground, then processed using an extraction kit (Qiagen^®^ AllPrep Fungal DNA/RNA/Protein Kit, Qiagen, Hilden, Germany) and following the manufacturer’s instructions with little modification. Quality of RNA was verified by demonstration of intact ribosomal bands in 1.5% agarose gel electrophoresis, in addition to 1.8–2.0 absorbance ratios (A260/280 and A260/230, respectively). First-strand cDNA was synthesized from 1 μg of DNA-free RNA using a reverse transcription mix (iScript™ Reverse Transcription Supermix, Bio-Rad Laboratories, Hercules, USA) following the manufacturer’s instructions. The cycle was set up as follows: one 5-min step at 25°C, a reverse transcription step at 46°C for 20 min, and a final step at 95°C for 1 min.

### Gene Expression Analysis of Laccase and MnP Genes

The expression level of the *Fomitiporia mediterranea* laccase (*Fmlcc*) and manganese peroxidase (*Fmmnp*) genes were determined by quantitative real-time PCR (qRT-PCR) using a CFX96 system (BioRad, Foster City, CA, United States). Primers for gene-specific amplification of *Fmmnp* were retrieved from the literature (Morgenstern et al., 2010), while that for gene-specific amplification of *Fmlcc* was designed using the Primer3 program^1^, retrieving template sequences published on the website of the National Center for Bioinformatic Information^2^. Primer specificity was checked by the Primer-BLAST tool on the NCBI webpage^3^. Transcription elongation factor 1 (*tef1*) was used as housekeeping gene to normalize the expression of target genes (Morgenstern et al., 2010). All the details on template sequences and specific primers are reported in Table 2.

**TABLE 2:** Gene accession numbers and sequences of primer pairs used for qRT-PCR.

Genes	Accession number	Primer sequences	Amplicon length (bp)
*Fmmnp1*	*HM480274.1*	*^†^Forward: 5′-ACGGCATTCCAAACGTCCATGAAG-3′*	*197*
		*^†^Reverse: 5′-GCACCAGGGTCCGTAGAAAGAGTA-3′*	
*Fmmnp2*	*HM480275.1*	*^†^Forward: 5′-GGCAATCAATGGTTGCAAACCAGC-3′*	*248*
		*^†^Reverse: 5′-AATCTGAGTCGCTTGTCCACCG-3′*	
*Fmmnp3*	*HM480276.1*	*^†^Forward: 5′-CGTCTTCAATCTGACTTCGCCCTC-3′*	*165*
		*^†^Reverse: 5′-GAGATCGGAGCAGTCAACGAGC-3′*	
*Fmlcc1*	*XM_007269683.1*	*Forward: 5′-TGGATCCGTGCTCAACCTTC-3′*	*206*
		*Reverse: 5′-AGTGCTAAGTCAACGCCTCC-3′*	
*Fmlcc3*	*XM_007269436.1*	*Forward: 5′-TTGGAGGCGGTACAGACAAC-3′*	*176*
		*Reverse: 5′-ACACAGTCCCAGCCAATCAG-3′*	
*Fmlcc4*	*XM_007269469.1*	*Forward: 5′-TCACTCGCATGAAGGAACCC-3′*	*177*
		*Reverse: 5′-GTTGAATTGGGTGGTCTGCG-3′*	
*Fmlcc7*	*XM_007261239.1*	*Forward: 5′-TCCTTTCCATCTGCACGGAC-3′*	*225*
		*Reverse: 5′-TCCACATCCTCAGCGAACAC-3′*	
*Fmlcc8*	*XM_007262111.1*	*Forward: 5′-AAGAGGCGGCGATGACTATG-3′*	*211*
		*Reverse: 5′-TTGACTTGTCGAACCTGGGG-3′*	
*Fmlcc9*	*XM_007263477.1*	*Forward: 5′-ACAAACTGGTCAAGGTGGGG-3′*	*223*
		*Reverse: 5′-AGCAAGGATGGAAGTGTCGG-3′*	
*tef1*	*AY885149.1*	*^†^Forward: 5′-TGGATTGCCACACTGCCCATATTG-3′*	*215*
		*^†^Reverse: 5′-GGTTTGCCTCATGTCACGCAC-3′*	

*Sequences accompanied by “†” were retrieved from published literature (Morgenstern et al., 2010), while the remaining ones were newly designed for this study.*

PCR reactions were carried out in a reaction mix containing 10 μl of iTaq Universal SYBR^®^ Green Supermix (Bio-Rad, CA, United States), 1 μl of forward and reverse primers (10 μM), and 15 ng of cDNA, in a final volume of 20 μl. Thermal cycling conditions were set up as follows: one 30-s cycle at 95°C, followed by 39 cycles at 95°C for 5 s and a step at 60°C for 20 s. All PCR amplicons were subjected to melt curve analysis from 55 to 95°C to evaluate their specificity, and a negative control was run without a cDNA template. The results obtained for each gene of interest were normalized to the expression of a housekeeping gene (*tef1*). Relative expression (2^–ΔΔCT^) compared to the mean of ΔCT of samples without wood was also calculated as described by Livak and Schmittgen (2001). Data are presented as means ± SE of three biological replicates, each one having three technical replicates per 96-well plate (Livak and Schmittgen, 2001).

### Statistical Analysis

Data normality and homoscedasticity were tested before running statistical analysis by the Shapiro–Wilk test and Bartlett test, respectively. The significance of differences in the dry weight of mycelial biomass grown in liquid culture and specific enzyme activity between Fmed strains was determined by a Tukey *post hoc* test after one-way ANOVA (*p* ≤ 0.05). A two-way ANOVA test (*p* ≤ 0.01) was performed for each polymer singularly, in order to establish if strain and grapevine variety significantly influence grapevine wood degradation. Differences between *Fmmnp* and *Fmlcc* expression of Fmed cultures grown in the presence of wood sawdust and cultures grown without wood sawdust were studied by Student’s *t*-test (*p* ≤ 0.05). Differences are highlighted by an asterisk when significant. The values reported are the averages of at least three replicates (*n* = 3) and are presented as mean values ± standard error (SE) of the mean. All analyses were performed in the R (3.6.3 version) programming environment.

## Results

**FIGURE 2:**
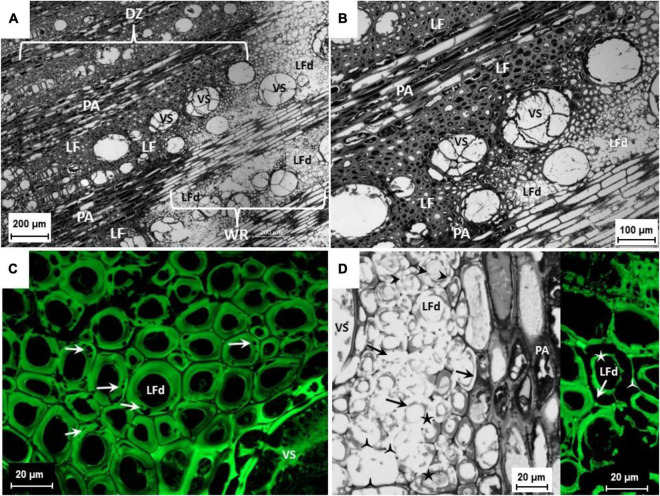
**(A,B)** Cross section of a symptomatic cv. Sauvignon blanc trunk. Lesion affected by white rot, vessels (VS) obstructed by tylosis; demarcation zone (DZ) identifiable by dark inclusions in the libriform fibers (LF) and parenchymal cells (PA) of the pith rays; white rot (WR) with decomposed libriform fibers (LFd). Bright-field Plan-Neofluar 5x **(A)** and bright-field Plan-Apochromat 10x **(B)**. **(C)** Cross section of a symptomatic cv. Sauvignon Blanc trunk. Lesion affected by white rot, vessels (VS) obstructed by tylosis; libriform fibers (LFd) with beginning of cell wall decomposition, S2 layer of the secondary cell wall with cavities caused by Fmed (arrows). Epi-FM image, excitation 460-488 nm, emission 500-557 nm; C-Apochromat 63x. **(D)** Cross section of a symptomatic cv. Riesling trunk. Lesion affected by white rot, vessels (VS) obstructed by tylosis; libriform fibers (LFd) with beginning of the cell wall decomposition, S2 layer of the secondary cell wall with cavities caused by Fmed (arrow heads) and advanced decomposition of the S2 layer (arrows), only middle lamella with primary cell wall and S1 layer (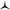) as well the S3 layer (★) of the secondary cell remained. Bright field, C-Apochromat 40x. On the right extract, libriform fibers (LFd) with advanced cell wall decomposition, S2 layer of the secondary cell wall totally decomposed, only middle lamella with primary cell wall and S1-layer (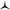) as well the S3 layer (★) of the secondary cell remained.

Epi-FM analyses performed on white-rotten wood portions highlighted evidence of cell degradation due to fungal enzyme activity, a common feature in white rot. Microscopic analysis revealed a distinct demarcation zone between the intact xylem and the white rot in which the cell lumina were discolored with dark inclusions. In contrast, the white-rotten region appeared lighter in color because of degradation of the lignocellulose (Figure 2A). The presence of tylosis in xylem vessel lumens denoted an active defensive activity of the vine in response to fungal development (Figures 2A,B). In the initial stage of the degrading activity, cavities and fissures occurred on the S2 layer of the secondary cell wall of the libriform fiber (Figure 2C), leaving apparently undamaged or slightly degraded only the middle lamella, the primary cell wall, and the S1 layer and S3 layer of the secondary cell wall (Figures 2C,D). In advanced decomposition, all layers of the cell wall of vessels, tracheids, and libriform fibers were decomposed including the lignified parts such as the middle lamella, the primary cell wall, and finally the walls of the parenchyma cells (Figure 2D). A soft rot-like cavity formation was also observed in some cases (Figure 2C). In *in vitro* fungal isolation of infected wood observed under the microscope, a high frequency of Fmed isolates (90%) was recorded. Other species isolated belonged mostly to *Penicillium* (6%) and only rarely to species in *Botryosphaeriaceae* (4%) (Table 3).

**TABLE 3:** Percentage of fungal taxa isolated from wood fragments sampled from grapevine wood tissue adjacent to the tissue area sampled for microscopy.

Fungal taxa	Incidence (%)
*Fomitiporia mediterranea*	90
*Penicillium* spp.	6
*Botryosphaeriaceae*	4

### Biomass Production

**FIGURE 3:**
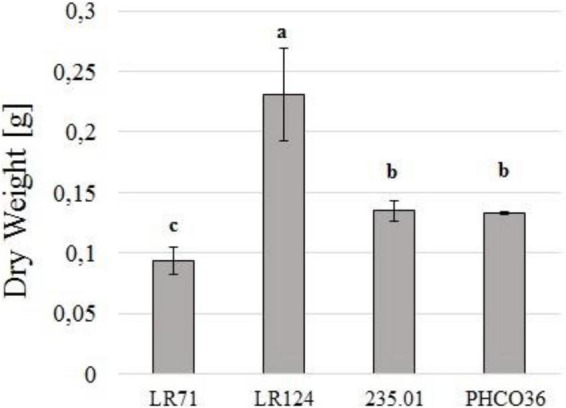
Dry weight of filtered liquid culture of 4 strains of Fmed (PHCO36, LR124, 235.01, LR71) grown at 28°C for 10 days in the dark. Cultures grown in 125 ml of Eriksson and Pettersson’s medium in the presence of cellulose and grapevine wood sawdust. Dried matter including everything was retained by 0.2-μm filtration. Statistical differences were calculated by ANOVA analysis (*p* ≤ 0.05), and letters above histograms indicate different statistical groups determined by Tukey’s test; error bars on histograms represent the standard error of the mean.

Strain LR124 produced the highest quantity of biomass, while LR71 was the strain which produced the least (−59.5% compared to LR124). Strains PHCO36 and 235.01 produced a comparable quantity of mycelial biomass (−42.4% and −41.5% compared to LR124, respectively) (Figure 3). Differences in growth performance were recorded also by measuring the protein content of the secretome. PHCO36 strain secretome contained 0.36 mg/ml of proteins after filtration, LR124 secretome 0.59 mg/ml, 235.01 secretome 0.54 mg/ml, and LR71 secretome 0.32 mg/ml.

### *In vivo* Wood Degradation and Residual Polymer Analysis

**FIGURE 4:**
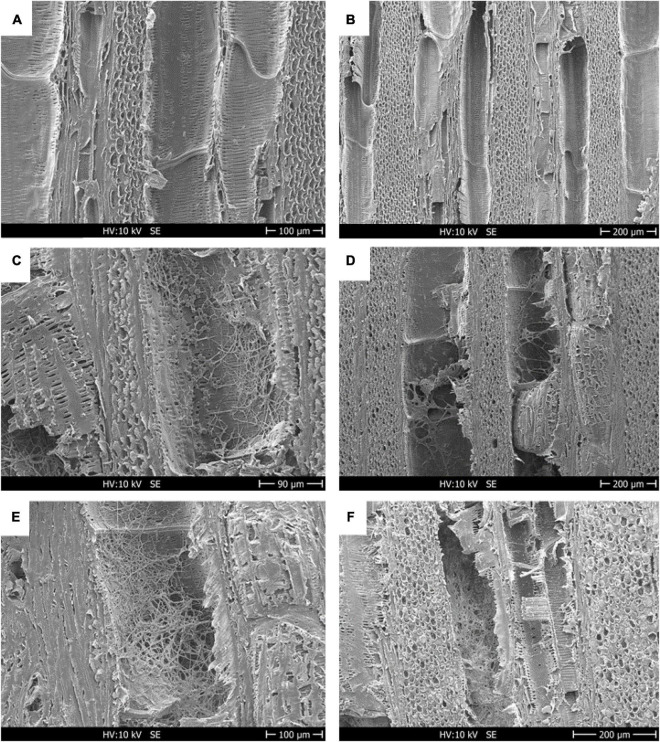
Scanning electron micrographs of cv. Gewurztraminer wood showing the longitudinal face colonized by the white-rot fungus Fmed. Before inoculation by an Fmed-solid culture plug, the wood structures were completely free of mycelium **(A,B)**; a diffuse colonization is appreciable after 30 days from inoculum **(C,D)**, and wider development of the fungus was detected after 90 days **(E,F)**. After 30 and 90 days, vessels and parenchyma cells were colonized by Fmed hyphae. Scale: **(C)** = 90 μm; **(A,E)** = 100 μm; **(B,D,F)** = 200 μm.

SEM analysis confirms the progress of Fmed colonization of the wood blocks subjected to degradation. Samples were sterilized before incubation with Fmed and, as shown in Figures 4A,B, all the structures were free of fungal mycelium. After 30 days’ incubation, the vessels were partly colonized by the Fmed mycelium (Figures 4C,D). 90 days after inoculation, all the wood structures were widely colonized by Fmed (Figures 4E,F). Based on these observations, it was assumed that the degradation of samples was carried out entirely by Fmed.

**FIGURE 5:**
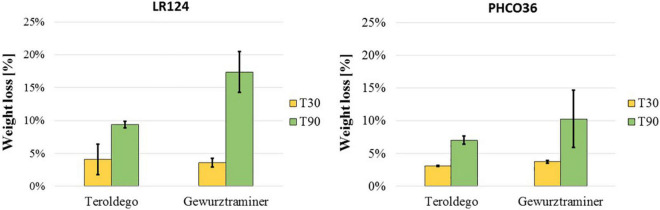
Grapevine wood weight loss after fungal degradation. Two grapevine varieties (cv. Teroldego and cv. Gewurztraminer) were compared. Weight loss is shown as the difference between wood blocks weight before and after degradation. For degradation, wood blocks were inoculated with 2 strains of Fmed (LR124 and PHCO36) and degraded at 28°C in the dark for 30 and 90 days. Error bars on histograms represent the standard error of the mean.

The weight loss of the wood blocks due to the degradation that occurred in the petri dishes, at 30 and 90 days, was calculated as the difference between the dry weight of the blocks measured before and after degradation. Weight loss after 30 days was less than 5% and comparable for both strains on both varieties. After 90 days’ degradation, both strains had been able to degrade the wood of the cv. Gewurztraminer more than the cv. Teroldego. LR124 degraded 9% of cv. Teroldego wood and 16% of cv. Gewurztraminer wood. The PHCO36 strain degraded 7% of cv. Teroldego wood and 10% of cv. Gewurztraminer wood. After 90 days, the LR124 strain had degraded more wood than the PHCO36 strain, as could already be observed after 30 days (Figure 5).

**FIGURE 6:**
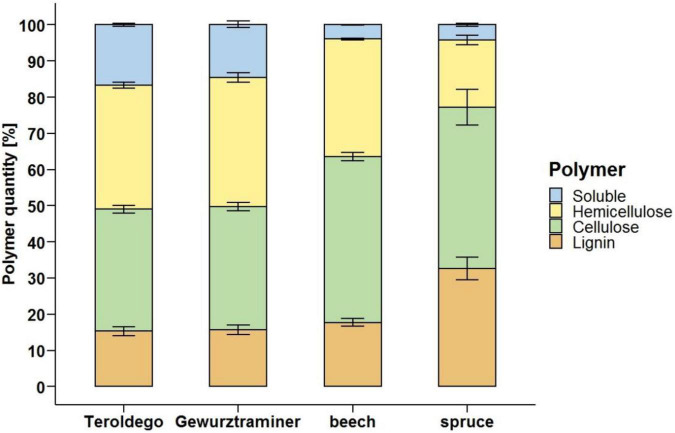
Grapevine (cv. Teroldego and cv. Gewurztraminer), beech, and spruce undegraded wood composition. Comparison of grapevine wood polymer composition to soft- (spruce) and hardwood (beech) profiles. Polymer content measures were performed on non-inoculated sound wood samples.

In addition to the loss in weight of the samples due to fungal degradation, the quantity of structural polymers of undegraded wood and the decrease of the different wood-forming polymers of wood incubated with Fmed were also analyzed. The lignin content of grapevine wood (15.3% cv. Teroldego and 15.7% cv. Gewurztraminer) was similar to the lignin content of beech (17.7%) and lower than the content in spruce (32.6%). In general, lignin represented the less abundant polymer in grapevine wood, similarly to hardwood, while cellulose and hemicellulose were present in grapevine wood in comparable proportions. The cellulose content of the grapevine wood tested (33.7% cv. Teroldego and 34% cv. Gewurztraminer) resulted lower than the beech (45.9%) and spruce (44.6%) content while hemicellulose represented 34.3 and 35.7% (cv. Teroldego and cv. Gewurztraminer, respectively) of grapevine wood, compared to 32.5% of hardwood and 18.6% of the softwood samples (Figure 6). The soluble fraction, i.e., pectin, proteins, sugars, and lipids, measured in grapevine wood represented a relevant percentage (16.7% cv. Teroldego and 14.7% cv. Gewurztraminer) compared to the quantity measured in spruce (4.2%) and beech (3.9%) wood.

**FIGURE 7:**
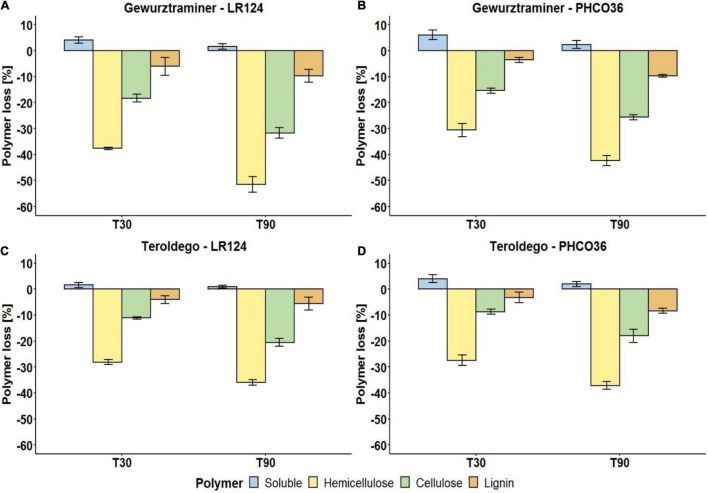
Degradation of wood polymers of two grapevine cultivars [cv. Gewurztraminer **(A,B)** and cv. Teroldego **(C,D)**] by two strains of Fmed [LR124 **(A,C)** and PHCO36 **(B,D)**] after 30 and 90 days. Degradation is presented as a percentage of polymer loss, compared with the untreated wood, 30 and 90 days after inoculation with the fungus. Error bars on histograms represent the standard error of the mean. ANOVA analysis (*p* ≤ 0.01) shows that strain and grapevine variety are factors which significantly influence all polymer degradation after 90 days.

Afterward, the decrease of soluble compounds, hemicellulose, cellulose, and lignin was evaluated on the incubated samples. In general, as shown in Figure 7, the content of soluble compounds increased because of the fungal enzyme activity on the wood components. Hemicellulose was the most degraded polymer by both Fmed strains. After 90 days, the LR124 strain degraded more hemicellulose than the PHCO36 strain on cv. Gewurztraminer wood (−51%) while on cv. Teroldego wood almost the same quantity was degraded by both strains (−36% LR124 and −37% PHCO36). On cv. Gewurztraminer wood, cellulose was degraded more by the LR124 strain (−32% after 90 days) than the PHCO36 strain (−26% after 90 days) as on cv. Teroldego wood (-21% LR124 and −18% PHCO36). Lignin was degraded more by the PHCO36 strain (−8%) on cv. Teroldego wood than the LR124 strain (-5.6%), while a comparable amount was degraded on cv. Gewurztraminer wood (−9.7% LR124 and −9.6% PHCO36). In general, after 90 days’ degradation, more hemicellulose was degraded on cv. Gewurztraminer wood (mean −47%) (Figures 7A,B), the variety that shows more symptoms of Esca, than on cv. Teroldego wood (mean −37%) (Figures 7C,D), the variety that shows fewer symptoms of Esca. The same observation can be made for cellulose (−27% on cv. Gewurztraminer and −19% on cv. Teroldego) and lignin (−9.6% on cv. Gewurztraminer and −7% on cv. Teroldego). Polymer degradation after 90 days of fungal growth was significantly affected by both fungal strain and grapevine variety (*p* ≤ 0.01).

### Enzyme Activity of Extracellular Protein Extracts

**FIGURE 8:**
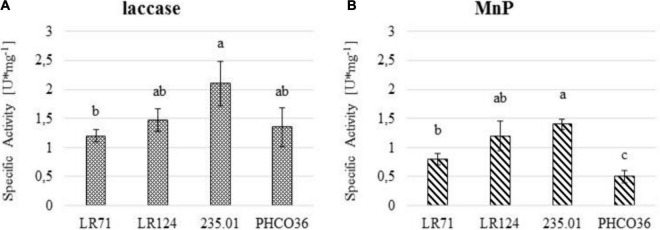
**(A)** Specific laccase and **(B)** manganese peroxidase (MnP) activity of 4 strains of Fmed measured by spectroscopy. Fmed liquid culture filtered secretome was used to measure the specific enzyme activity. U/mg unit represents 1 μmol of substrate oxidized per minute per mg of protein. ANOVA was performed to determine significant differences (*p* ≤ 0.05), and Tukey’s test was performed for statistical group identification. Error bars on histograms represent the standard error of the mean.

Laccase and MnP-specific activity values (Figure 8) evidence that in the tested strains, 235.01 showed the greatest overall specific enzyme activity. The specific laccase and MnP activities of the LR124 strain were among the highest (Figures 8A,B), lower than those of strain 235.01, but not statistically different. The specific laccase activity of strain LR71 was the lowest, while the weakest specific MnP activity was recorded for PHCO36. Overall, the enzyme activity of the PHCO36 strain was the weakest despite it not being statistically different from that of the LR71 strain in the laccase assays. Generally, laccase activity was higher than MnP activity for all tested strains.

### Gene Expression

**FIGURE 9:**
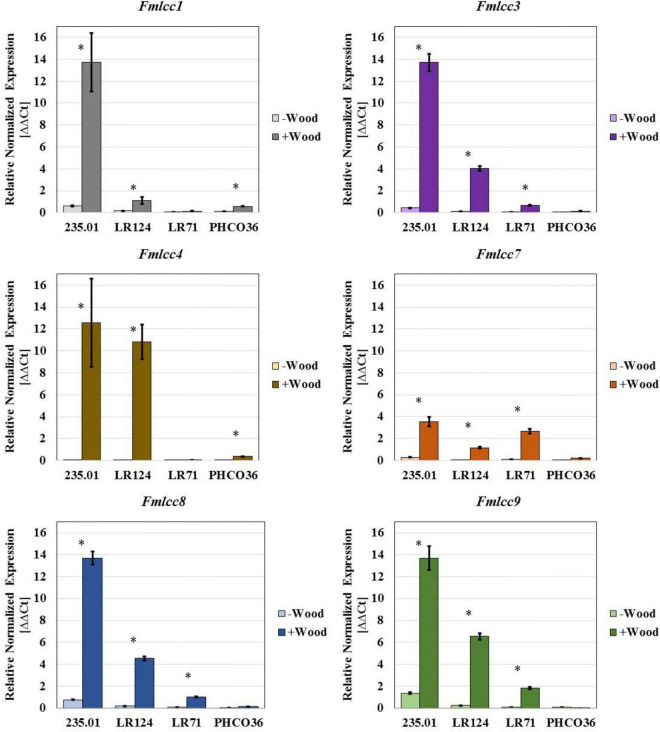
Gene expression of six laccase isoforms measured in 4 different strains of Fmed. Data are expressed as relative normalized expression of genes with cv. Gewurztraminer wood sawdust (+ Wood) and without sawdust (–Wood) compared to the mean of ΔCT of samples grown without wood sawdust. The asterisk denotes significant difference in Student’s *t*-test (*p* ≤ 0.05) between gene expression with and without wood sawdust. Error bars on histograms represent the standard error of the mean.

**FIGURE 10:**
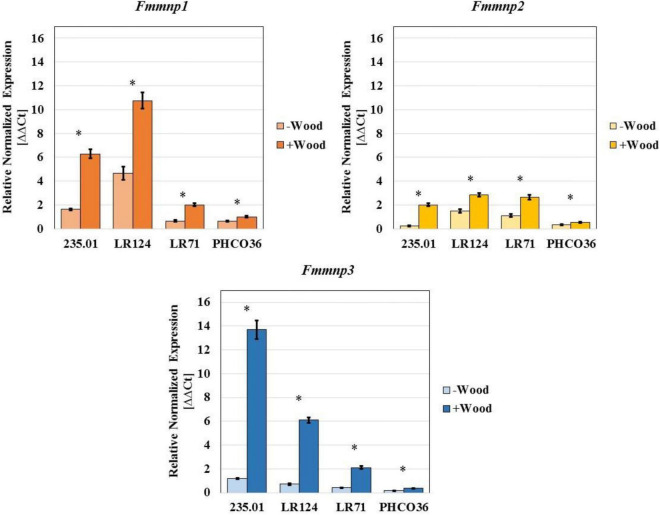
Gene expression of three manganese peroxidase isoforms measured in 4 different strains of Fmed. Data are expressed as relative normalized expression of genes with cv. Gewurztraminer wood sawdust (+ Wood) and without sawdust (–Wood) compared to the mean of ΔCT of samples grown without wood sawdust. The asterisk denotes significant difference in Student’s *t*-test with *p* ≤ 0.05 between gene expression with and without wood sawdust. Error bars on histograms represent the standard error of the mean.

With the aim of supporting a specific enzyme activity test, an *in vitro* assay was performed by qRT-PCR to assess the differential ability to express laccase- and MnP-coding genes, in the presence or absence of grapevine wood sawdust (Figures 9, 10). The relative normalized expression of laccase- and MnP-coding genes, regardless of wood sawdust presence, confirms the intra-specific differences observed by enzyme activity tests. However, the data showed how the presence of wood sawdust induces an upregulation of all genes, without changing the trend in basal expression. The laccase-coding gene less affected by the presence of wood sawdust resulted as *Fmlcc7*. Strain 235.01 showed the highest level of transcript abundance of all the laccase-encoding genes compared to the other strains, when cultivated in the presence of wood sawdust. The expression level of the laccase-coding gene by the LR124 strain resulted higher than LR71 for all isoforms except *Fmlcc7*. The PHCO36 strain showed the weakest *Fmlcc* and *Fmmnp* expression in general: 4 laccase-encoding genes of the PCHO36 strain were not upregulated by the presence of sawdust, compared to their basal expression. Moreover, the data show how *Fmlcc3*, *Fmlcc8*, and *Fmlcc9* seem to be the most upregulated laccase-encoding isoforms, in the presence of sawdust, considering all strains (Figure 9).

Concerning MnP-encoding genes, strain LR124 showed the highest level of *Fmmnp1* and *Fmmnp2* transcript abundances in the presence of wood sawdust, while strain 235.01 showed the highest level of expression of the *Fmmnp3* gene. Relevant variations in the expression of all the tested MnP-encoding genes were observed: the *Fmmnp1* gene and *Fmmnp3* gene majorly contributed to *Fmmnp* expression compared to *Fmmnp2*, and as recorded for laccase-encoding genes, the presence of wood sawdust induced a higher level of gene expression (Figure 10). As recorded for laccase-encoding genes, the PHCO36 strain was shown to be the strain with the weakest MnP-encoding gene expression. For each tested gene, the significance of the difference in gene expression between culture grown with and without sawdust was statistically confirmed (*p* < 0.05).

## Discussion

The results obtained in the present study show which wood cellular structures are mainly concerned by the degrading activity of Fmed. The epi-fluorescence microscopy analysis denoted a degrading pathway between selective white-rot and soft-rot characteristics. The degradation of grapevine wood was replicated *in vitro*, and the decrease in each wood-constituent polymer was measured by incubating sterilized vine wood blocks with different Fmed strains: grapevine variety and Fmed strain were shown to be factors influencing the degrading process. Based on wood-constituent consumption, as all the measured polymers were degraded simultaneously (Figure 7), the classification as selective white rot cannot therefore be confirmed. Furthermore, the intraspecific variability in laccase and MnP enzyme activity, and finally the gene regulation of these enzymes, was documented. Microscopy observations on the structure of vine wood cells naturally infected by fungal decay showed the common signs of white-rot biodegradation also described by other authors (Eriksson et al., 1980; Schwarze, 2007; Liew et al., 2011). All the woody structure alterations observed in this study were mainly attributed to Fmed after classic fungal isolation on an artificial substrate, which confirmed a very high incidence of Fmed in the rotten vine wood. High incidence of Fmed in the grapevine decayed wood is commonly reported (Larignon and Dubos, 1997; Maher et al., 2012). Fmed is a white-rot fungus holding the most efficient wood-degrading enzymatic pool (Floudas et al., 2012; Riley et al., 2014). The observed alteration in grapevine wood structure, mainly at secondary cell wall level and on the median lamella, due to the activity of Fmed, possibly suggests a selective white-rot behavior. In fact, when selective delignification occurs, the decay process starts from the cell membrane side outward and can also affect the middle lamella regions of adjacent cells, leaving fibers weakly structured (Daniel, 1994, 2014). Moreover, the decomposition pattern observed also displays some characteristics of soft rot degradation. The diamond-shaped cavities highlighted by the epi-fluorescence microscopy observations on the S2 layer of secondary cell walls are similar to those formed by soft rot, namely, type I soft rot degradation (Anagnost et al., 1994; Schwarze, 2007; Daniel, 2014). The activity of the Ascomycota species in the formation of the soft rot decay cannot be excluded, although very few colonies other than Fmed have been isolated from the sampled wood. The decay pattern observed on the S2 layer has previously been reported for some other *Basidiomycetes*, but it is known among several wood-degrading Ascomycetes (Schwarze et al., 1995; Martínez et al., 2005). The analysis of the residual polymers carried out on grapevine wood samples after 30 and 90 days’ degradation showed that hemicellulose was the wood component most extensively degraded, followed by cellulose and lignin, in that order. The results obtained suggest that as much as 50% of hemicellulose, 30% of cellulose, and 10% of lignin of grapevine wood could be mineralized after 90 days under optimal conditions. The polymer degradation ratio varied significantly between strain and grapevine varieties. Residual polymer analysis showed a non-negligible cellulose consumption, which is uncommon for selective white rot. In selective white rot, lignin and hemicellulose are “preferentially” (selective white rot is also named preferential white rot) degraded and a large concentration of cellulose is normally left (Blanchette et al., 1987; Eriksson et al., 1990). Thus, based on the wood polymer consumption, Fmed polymer-relative consumption appears to be more associable with simultaneous white rot than with selective white rot (Blanchette, 1991; Daniel, 2003). Otherwise, this may have occurred because *in vitro* assays reproduce an unreal environment with a high availability of oxygen and a high moisture level. Under aerobic conditions (a quite rare circumstance inside a woody plant (*sensu lato*) solid trunk), white-rot fungi can completely mineralize lignin and wood polysaccharides through the production of hydrogen peroxide as a extracellular oxidant, to CO_2_ and H_2_O (Sánchez, 2009; Daniel, 2014). Also the high moisture level of the substrate in the petri plate could have influenced the fungal degrading activity (Brischke and Rapp, 2008). This analysis also supplemented data on the polymer constitution of grapevine wood, which had only been reported once in the literature (Agrelli et al., 2009). Afterward, intraspecific differences among the strains were first highlighted by the analysis of laccase and MnP activity and then confirmed *via* gene expression of 6 laccase- and 3 MnP-encoding genes. The reported high number of laccase and MnP isoforms could be potentially explained by the high presence of repetitive sequences in the Fmed genome. In fact, it is generally accepted that transposable elements and microsatellites are responsible for rearrangements and gene mutation in *Basidiomycetes* (Castanera et al., 2017). Laccase activity was slightly predominant compared to MnP activity according to the results obtained on different white-rot *Basidiomycetes* (Mathieu et al., 2013). The white-rot wood-degrading enzymatic pool, the oxidative and extracellular ligninolytic system which depolymerizes lignin, is solidly represented by laccase, MnP, and LiP. Laccases are phenoloxidase that oxidize phenolic and non-phenolic compounds in the presence of specific mediators (Bourbonnais et al., 1995; Call and Mücke, 1997; Gianfreda et al., 1999; Majcherczyk et al., 1999). On phenolic compounds, one-electron oxidation generates phenoxy-free-radical products, which can lead to polymer cleavage. MnPs catalyze the Mn(II) oxidation to Mn(III) after its chelation by organic acid, in the presence of H_2_O_2_ (Kuwahara et al., 1984; Glenn and Gold, 1985). Mn(III) oxidated by MnPs can oxidize phenolic compounds but not non-phenolic units of lignin (Pérez et al., 2002). Upon completion, the hydrolytic system of fungi, responsible for cellulose and hemicellulose degradation, is formed by a wide range of enzymes: hemicellulose biodegradation needs the joint action of several enzymes such as xylan esterases, ferulic and p-coumaric esterases, α-l-arabinofuranosidases, and α-4-O-methyl glucuronidases (Kirk and Cullen, 1998), while cellulases, namely, endoglucanases (EGs) and cellobiohydrolases (CBHs), synergically degrade cellulose. The degradation of lignin-based cellular structures observed by epi-fluorescence microscopy and the polymer loss measured in this study can therefore both be attributed to the enzymes mentioned above (Goodell, 2020). LiP activity was not considered in this study, based on evidence that excludes the presence of LiP-encoding genes in the Fmed genome (Floudas et al., 2012). In order to gain further details on the fine regulation of grapevine wood degradation process by Fmed, the gene expressions of MnP and laccase-encoding genes were studied. According to the literature, the use of a gene expression stimulator (sawdust in our case) allowed an upregulation of studied genes (Mansur et al., 1998; Giardina et al., 2000; Ohga and Royse, 2001; Hakala et al., 2006; Sakamoto et al., 2009). Regardless of wood sawdust presence, the results highlight a basal expression of the MnP-encoding genes higher than that of laccase. Furthermore, the expression of the targeted genes highlighted remarkable intraspecific variability, in line with the observed genetic population variability (Pollastro et al., 2001; Jamaux-Despréaux et al., 2003). The measurement of the enzyme activity *in vitro* and the correlated gene expression are consistent with results previously obtained by other authors (Abou-Mansour et al., 2009; Morgenstern et al., 2010; Cloete et al., 2015a). In conclusion, if we assume that the pores of the cell walls may not be large enough to allow the studied enzymes to reach the core of the secondary cell wall, as proposed by other authors for other pathosystems, the cavitation phenomena observed in the S2 layer of the secondary cell walls cannot be justified solely by the activity of the enzymes (Evans et al., 1991, 1994; Flournoy et al., 1991, 1993; Paice et al., 1995). Therefore, a lack of link between the degradative potential of the tested enzymes and the evidence observed *via* microscopy emerges as a general result from this multidisciplinary experiment. Based on this, we can thus hypothesize that a preliminary non-enzymatic activity, i.e., the Fenton-type chemical reactions, allowed the enlargement and the damage of the cell wall pores in order to allow the enzymes to produce the observed degradation effects (Goodell et al., 1997, 2017, 2019; Arantes and Milagres, 2009; Moretti et al., 2019). The role of low molecular weight compounds (LMWC) postulated by these authors is also crucial in other white-rot fungi, since they can also act as mediators of specific lignin-degrading enzymes such as laccase and MnP (Faison et al., 1986; Jellison et al., 1991; Dutton et al., 1993; Eggert et al., 1996; Ten Have and Teunissen, 2001; Arantes and Milagres, 2007). During this preliminary phase, bacterial activity could also potentially influence wood attack (Haidar et al., 2021). Considering what was observed in this study and taking into account the information just introduced, further investigations on the wood-degrading mechanisms caused by *Fomitiporia mediterranea*, as well its interaction with the wood microbiota, are required to shed light on factors possibly involved in foliar symptom expression.

## Conclusion

Over the years, several studies have focused on putative factors that can trigger the Esca-associated leaf stripe symptoms: metabolomic, metagenomic, biochemical, and chemical approaches have been adopted. Much evidence has been presented over the last few years, but even today many aspects of the ECD continue to be unclear. The complex etiology attributed to the leaf symptom development, namely, GLSD, makes it even more difficult to determine which factors may actually influence the generation of symptoms. This study highlighted the most important metabolic aspects of one of the main fungal species associated with the Esca complex. The role of *Fomitiporia mediterranea* in Esca diseases has recently been investigated in more depth, and the results presented here not only strengthen knowledge on the enzymatic processes that lead to the degradation of vine wood but also pave the way for a new line of research, focused on the processes of synergistic enzymatic and non-enzymatic degradation of wood, which could be involved in triggering the foliar symptoms.

## Data Availability Statement

The original contributions presented in the study are included in the article/supplementary material, further inquiries can be directed to the corresponding author/s.

## Author Contributions

AP contributed to the study design, conducted the laboratory trials, analyzed the data, and wrote the original manuscript. SM contributed to the study design, assisted with the laboratory trials, and improved the manuscript. CP assisted the laboratory trials. EB conducted the microscopy trials. H-HK provided the naturally infected wood samples and revised the manuscript. EG revised the manuscript. LM revised and polished the manuscript. SF and CB designed the study. All authors contributed to the manuscript and approved the submitted version.

## Conflict of Interest

The authors declare that the research was conducted in the absence of any commercial or financial relationships that could be construed as a potential conflict of interest.

## Publisher’s Note

All claims expressed in this article are solely those of the authors and do not necessarily represent those of their affiliated organizations, or those of the publisher, the editors and the reviewers. Any product that may be evaluated in this article, or claim that may be made by its manufacturer, is not guaranteed or endorsed by the publisher.

## Footnotes

1
https://bioinfo.ut.ee/primer3-0.4.0/

2
http://www.ncbi.nlm.nih.gov/

3
https://www.ncbi.nlm.nih.gov/tools/primer-blast/
